# Impact of Image Resolution on Deep Learning Performance in Endoscopy Image Classification: An Experimental Study Using a Large Dataset of Endoscopic Images

**DOI:** 10.3390/diagnostics11122183

**Published:** 2021-11-24

**Authors:** Vajira Thambawita, Inga Strümke, Steven A. Hicks, Pål Halvorsen, Sravanthi Parasa, Michael A. Riegler

**Affiliations:** 1 Simula Metropolitan Center for Digital Engineering, 0167 Oslo, Norway; inga@simula.no (I.S.); steven@simula.no (S.A.H.); paalh@simula.no (P.H.); michael@simula.no (M.A.R.); 2 Faculty of Technology, Art and Design (TKD), Oslo Metropolitan University, 0167 Oslo, Norway; 3 Swedish Medical Group, Department of Gastroenterology, Seattle, WA 98104, USA; vaidhya209@gmail.com

**Keywords:** image resolution, convolutional neural networks, endoscopic images

## Abstract

Recent trials have evaluated the efficacy of deep convolutional neural network (CNN)-based AI systems to improve lesion detection and characterization in endoscopy. Impressive results are achieved, but many medical studies use a very small image resolution to save computing resources at the cost of losing details. Today, no conventions between resolution and performance exist, and monitoring the performance of various CNN architectures as a function of image resolution provides insights into how subtleties of different lesions on endoscopy affect performance. This can help set standards for image or video characteristics for future CNN-based models in gastrointestinal (GI) endoscopy. This study examines the performance of CNNs on the HyperKvasir dataset, consisting of 10,662 images from 23 different findings. We evaluate two CNN models for endoscopic image classification under quality distortions with image resolutions ranging from 32 × 32 to 512 × 512 pixels. The performance is evaluated using two-fold cross-validation and F1-score, maximum Matthews correlation coefficient (MCC), precision, and sensitivity as metrics. Increased performance was observed with higher image resolution for all findings in the dataset. MCC was achieved at image resolutions between 512 × 512 pixels for classification for the entire dataset after including all subclasses. The highest performance was observed with an MCC value of 0.9002 when the models were trained on the highest resolution and tested on the same resolution. Different resolutions and their effect on CNNs are explored. We show that image resolution has a clear influence on the performance which calls for standards in the field in the future.

## 1. Introduction

Research communities have put great efforts towards the automation of computer-aided diagnostic tools with the ability to detect and classify a variety of different endoscopy findings. Consequently, automated evaluations of endoscopy-related lesion detection can be used to augment the performance of endoscopists [1,2,3].

In recent years, Convolutional Neural Networks (CNNs) have emerged as one of the most successful image classification models [4]. In general, a CNN image classifier consists of a combination of convolutional layers, pooling layers, fully connected layers, and the soft-max layer. How many layers and how they are combined depends on the architecture of the network. A CNN takes an image as input, learns the image’s spatial information, and creates feature maps which are the input for the following layers [5]. Hence, spatial-visual information is an important component on which improved performance can be achieved. Therefore, the quality of the images and videos used during the development and application of these methods is a crucial factor. Several factors can cause the quality of collected images to vary significantly, examples include but are not limited to: the operator’s expertise, the type of endoscope used, physical barriers, and other disturbances. One can also see a large variation from high quality images to low quality ones in real world applications. This also depends on the equipment: for example, newer generations of smartphones take high quality and resolution pictures, whereas images in medical fields often cannot be assumed to be of high quality (due to old equipment, software, or lack of storage space for high quality data). Image quality factors, such as resolution, noise, contrast, blur, and compression, affect the visual information contained in the images [6]. Although the immediate visual information does not necessarily vary significantly, the details preserved in the visual information (e.g., fine vessels, the structure of the polyp surface) can vary drastically with the reduction of image resolution.

In general, the resolutions for training CNNs usually range between 64 × 64 and 256 × 256. Previous studies on the role of image resolution in chest radiographs show that image resolution impacts CNN performance [7]. In this study by Sabottke et al., it is shown that better model performance was achieved with lower input image resolutions. While this might seem paradoxical, a lower number of input variables or features is often desirable in applications of deep architectures. This is because lowering the number of parameters that need to be optimized reduces the risk of model overfitting [8].

Based on these prior results and to reduce processing time and resource requirements, the images today are typically down-sampled to a fraction of the original resolution. However, extensive reduction of the image resolution eventually leads to the elimination and loss of important information in the image that is used for the classification. Especially, if the important information lies hidden in small details, such as blood vessels, pit appearance, the surface of the lesion, and other patterns of the findings. Furthermore, there is an inherent trade-off in CNN implementations, as a graphics processing unit (GPU)-based optimization can have limitations where higher image resolution can reduce the usable batch size (number of samples given to the neural network per training iteration), which can, in turn, impact the model performance. Determining the optimum image resolution for different endoscopy related image-based lesion detections and characterizations is thus an important question that remains to be answered. The primary goal of this article is to perform an experimental study of varying image resolutions and assess its effects on the performance of CNN-based image classifiers related to gastrointestinal (GI) endoscopy.

## 2. Methods

**Figure 1:**
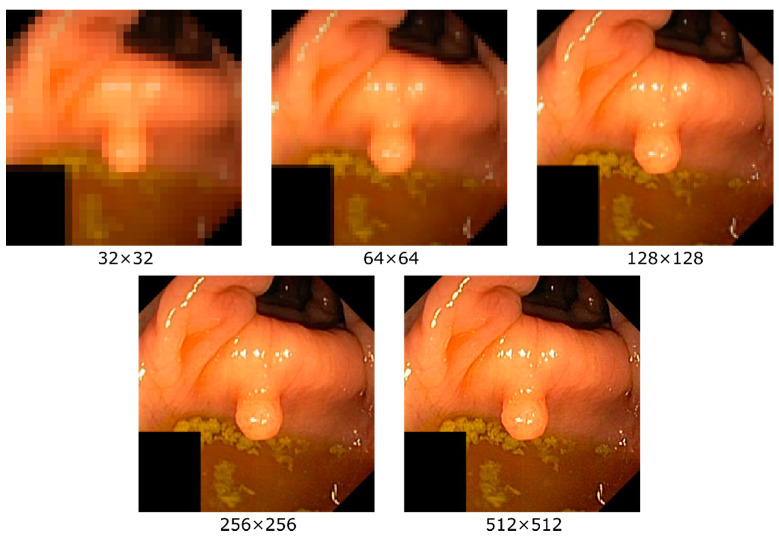
Examples of an image with the different resolutions used for the experiments in this article. Clear differences in the level of details that are detectable can be observed. Note that for this figure all resolutions are re-scaled to the same size to show quality differences.

To study the effect of different image resolutions on the performance of a CNN model, we use two well-established deep learning architectures on the publicly available HyperKvasir dataset consisting of 10,662 endoscopic images from 23 different findings [9]. We measure the classification performance of two CNN architectures, a residual neural network architecture [10] (ResNet) and a dense neural network architecture [11] (DenseNet), on images of different findings that can occur during endoscopy with a varying level of resolution. These two CNN architectures are selected based on the performance shown in the study [12], which has selected the two networks based on the state-of-the-art performance of top accuracies for the ImageNet [13] dataset. The CNN architectures are initialized with the ImageNet [13] weights as provided by PyTorch. Then, both models are trained using different resolutions where we saved the best checkpoints for each resolution. The resolutions we study are 32 × 32, 64 × 64, 128 × 128, 256 × 256, and 512 × 512 (the highest common resolution for all images within the dataset is 512 pixels, thus being the upper limit). An example of the effect of different resolutions on an image is given in Figure 1. One can easily see that the level of details perceptible in the image increase with higher resolution, i.e., as expected, details are lost when down-sampling. In addition to training and testing with the same resolution, we also perform experiments where the resolution between the training and testing datasets is varied (e.g., a model is trained on 32 × 32 and tested on 64 × 64, 128 × 128, 256 × 256, and 512 × 512). For all experiments, the same configuration and hyperparameters are used, i.e., as set by ImageNet [10,11].

To evaluate performance, we perform all experiments using two-fold cross-validation (50:50 data splits) and report the average score of the two folds. The split of the folds is done randomly at the beginning of the experiments and remains the same across the different resolutions. The metrics used to evaluate the performance are precision, sensitivity (also called recall), F1-score, and Matthews correlation coefficient (MCC). Since the numbers of images per class of the datasets are not equally distributed (which is common for medical datasets), we choose to bias our precision, sensitivity, and F1-score metrics towards the least populated classes, which is more relevant for medical applications. Thus, we report macro averaged results for these three metrics [14], but not for MCC since it is robust against bias in the classes [15].

### 2.1. Experimental Setup

We use the HyperKvasir dataset [9], which contains 10,662 images depicting 23 different findings of the gastrointestinal (GI) tract (the findings in the dataset contain anatomical landmarks, pathological findings in the lumen, colon polyps, Barrett’s esophagus, ulcerative colitis, etc.). No duplicate images are included in the dataset, i.e., each finding is only represented by a single frame, giving the data a large diversity. A complete overview of all findings in the dataset can be found in Table 1.

The dataset consists of images with different resolutions ranging from 720 × 576 to 1920 × 1072 pixels. The maximum resolution used in our experiments is 512 × 512, which is an optimal combination of the maximum shared resolution between all samples in the dataset, the used network architectures, and the available GPU memory. The CNNs are implemented using the Pytorch framework version 1.6 and Python version 3.8. The used hardware is an NVIDIA DGX-2 machine using NVIDIA V100 Tensor Core GPUs with the Ubuntu 18.04 operating system and CUDA version 10.1. 

### 2.2. Convolutional Neural Networks

In total, we trained 20 different models (two models × two folds × five different resolutions), which are used to obtain results for 100 different resolution combinations. As mentioned earlier, we perform two-fold cross-validation and switch the train and test dataset for the different folds. The precision, sensitivity, F1-score, and MCC are calculated using macro and micro averaging.

We use the two most basic CNN architectures from the five methods discussed in the paper [16]. The first method uses pre-trained (using ImageNet) DenseNet-161 and the second method ResNet-152 to predict 23 classes. We select these basic architectures over the more complex ones because our aim is not to demonstrate the best well-performing methods, but rather the effect the input image resolution has on the performance. In both cases, we use cross-entropy loss and stochastic gradient descent as loss function and optimizer, respectively. We use an initial learning rate of 0.001 and reduce it by a factor of 10 when the models do not show any progress in validation performance for 25 consecutive epochs, using the learning rate scheduler from Pytorch [16]. For our final predictions, we use the best-scoring model after an early stopping conditioned upon a learning rate of 10e−6.

## 3. Results

**Figure 2:**
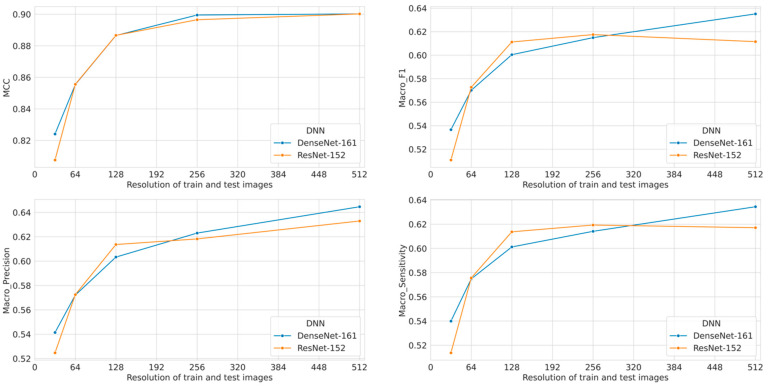
Comparison of MCC, macro F1, macro precision, and macro sensitivity when the models are trained and tested with the same input resolution.

Table 2 shows the results of the performance of the CNN algorithms for endoscopic image classification for varying image resolutions. The performance of the CNNs is reported in terms of precision, sensitivity, F1-score, and MCC for the DensNet-161 and ResNet-152 models. The presented numbers are the average over both folds in the cross-validation. We observe that increasing the resolution leads to increased performance measured in almost all metrics for both models. There is a slight decrease in sensitivity and F1-score in ResNet-152 for the highest resolution (512 × 512) compared to the lower resolution (256 × 256), but taking the MCC value into account there is an overall improvement. Comparing the two models, we see that they perform and behave quite similarly as noted by the MCC score which is almost the same. Figure 2 depicts the increase in performance as measured by MCC, macro F1, macro precision, and macro sensitivity with increased image resolution.

**Figure 3:**
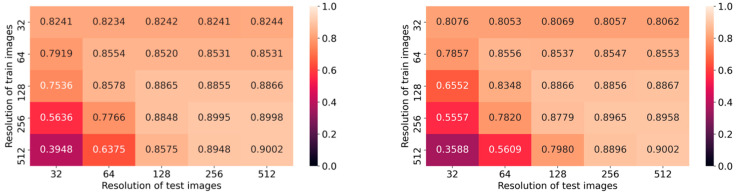
Averaged MCC from two-fold cross-validation as confusion matrices. **Left** is from DenseNet-161 and **right** is from ResNet-152.

Additionally, we analyze the impact of using different input resolutions on DNN models trained with a fixed resolution and reporting the performance metric on MCC. Average values from the two folds of DenseNet-161 and ResNet-152 are plotted as confusion matrices in Figure 3. The larger the difference in the resolution, the lower the performance. We also observe a clear correlation between different train and test resolutions on both axes in the confusion matrices, for both architectures. Furthermore, we have analyzed the time consumed by the models to perform predictions, and the results are tabulated in Table 3. 

**Figure 4:**
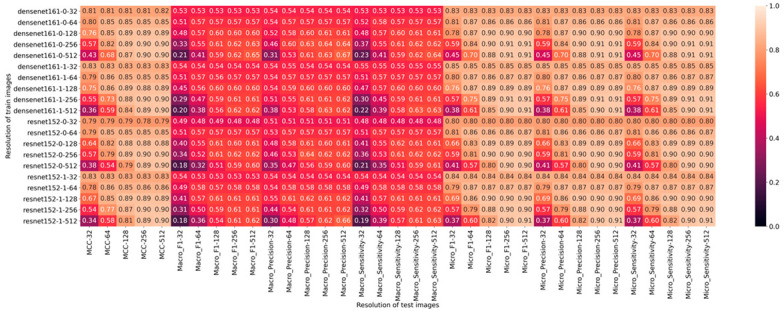
A complete overview of all obtained results including the macro and micro average for precision, sensitivity, and F1-score.

A complete overview of all obtained results including the macro and micro average for precision, sensitivity, and F1-score is shown in Figure 4.

## 4. Discussion

We evaluate the impact of low resolution on the performance of endoscopic image classification using two CNNs, i.e., ResNet-152 and DenseNet-161. Our findings are consistent with prior studies evaluating the role of image resolution on the performance of lesion detection, and classification accuracy in radiology and ophthalmology [7].

Primarily, low image resolution can significantly decrease the classification performance of CNNs, as shown in Figure 2. This is true even if the decrease in the image resolution is relatively small: A noticeable drop in performance is still observed for the lowest considered decrease in resolution, arguably difficult to spot with a naked eye in many cases. For endoscopic deep learning applications, particularly those focused on subtle lesions such as sessile serrated adenomas, dysplasia in Barrett’s esophagus, etc., even small performance changes can potentially have significant effects on patient care and outcomes. In contrast, Table 3 shows that an increasing image resolution does not have a huge effect on the prediction speed in the inference stage. Then, having high-resolution images with deep learning methods has better advantages when we cannot see any considerable performance drops. 

For mixed resolution cases, we observe that up-scaling from lower resolution results in a higher performance loss than down-scaling from higher resolutions. This suggests the need for images in GI datasets to be collected in high resolution, given that down-scaling is easy, while up-scaling to the original resolution is (given the tools available at the time of writing) impossible. 

Currently, CNNs usually operate on low to mid-level resolutions (256 × 256 and lower). In the field of GI endoscopy, different deep learning applications have employed many different image resolutions that can be compared to the different image resolutions we used in our experiments. However, unfortunately, the details of the resolution of the images and how these models perform in varying resolution is not always mentioned. For example, in the paper by Wang et al. [3], they mention that among low quality images, the sensitivity of polyp detection is significantly lower. Given that real-time endoscopy in the community can have varied image resolutions, it has to be borne in mind that these algorithms which perform excellently in controlled studies using endoscopic images with high resolution might perform poorly in real life.

Higher-resolution datasets might require new methods, architectures, and hardware. As hardware improvements and algorithmic advances continue to occur, developing deep learning applications for endoscopy at higher image resolutions becomes increasingly feasible. Nevertheless, although the full potential of high-resolution datasets might not be exploitable yet, it is evidently important to collect data with the highest resolution possible.

One limitation of our present work was that, due to graphics processing unit memory constraints, we fixed the batch size at eight for all models as our hardware was not capable of training high-resolution models at larger batch sizes. However, as hardware advances make graphics processing units with larger amounts of random access memory increasingly available, there is an opportunity for obtaining better performance from high image resolution models with larger batch sizes.

Several directions for further research can be envisioned: First of all, the use of technology such as super-resolution remains unexplored in the context of endoscopic images. It is likely that given future improvements in the quality of super-resolution methods, it will be possible to further reduce the negative impact low-resolution images have on current classification performance. Further research exploring the impact of image resolution on specific subclasses of the images (e.g., Barrett’s esophagus and Ulcerative colitis) was not done and is beyond the scope of this paper. However, we provide the code and documentation of the system used in the current study on GitHub (https://github.com/vlbthambawita/Endoscopy_Res_vs_DL (accessed on 19 November 2021)) to promote reproducibility. 

For future work, an important consideration is a possible trade-off between image size on one hand, and the time needed both for training the CNN model making new predictions on the other. Usually, we can observe, as shown in another study [17], that the lower the resolution, the faster the latter two. In addition, the highest resolutions (above HD) require either complicated training paradigms (e.g., distributed learning) or specific hardware, which are not standard or widely available yet.

## 5. Conclusions

In this paper, we propose a methodology to evaluate the effect of image resolution on the performance of CNN-based image classification by using a standard image dataset HyperKvasir. The experimental results and analysis conclude that the performance of the classifier is mainly dependent upon visual information and the resolution of images. A decrease in image resolution decreases the performance of the CNN-based image classification as quantified by lower MCC, F1, precision, and sensitivity results. Therefore, given that higher image resolutions lead to better performance of the CNN models, the current trend of reducing the resolution for faster processing needs to be reconsidered in the future in the realm of GI endoscopy computer-aided diagnosis. Details regarding the characteristics of the image resolution and the performance of the models at different resolutions should be mentioned in research papers to facilitate realistic expectations of such technology. Moreover, minimum standards for image resolution as it pertains to GI images need to be considered. 

## Figures and Tables

**Table 1:** Statistic of the dataset used for the experiments. Split 0 and Split 1 represent two folds used in our experiments.

Class	Split 0	Split 1	Total
Barrett’s Esophagus	20	21	41
BBPS-0-1	323	323	646
BBPS-2-3	574	574	1148
Dyed-lifted-polyps	501	501	1002
Dyed-resection-margins	494	495	989
Hemorroids	3	3	6
Ileum	4	5	9
Impacted-stool	65	66	131
Normal-cecum	504	505	1009
Normal-pylorus	499	500	999
Normal-z-line	466	466	932
Esophagitis-LA grade A	201	202	403
Esophagitis-LA grade B-D	130	130	260
Colon Polyp	514	514	1028
Retroflex-rectum	195	196	391
Retroflex-stomach	382	382	764
Short-segment-Barrett’s	26	27	53
Ulcerative colitis-Mayo score 0–1	17	18	35
Ulcerative colitis 1–2	5	6	11
Ulcerative-colitis-Mayo 2–3	14	14	28
Ulcerative-colitis-grade-1	100	101	201
Ulcerative-colitis-grade-2	221	222	443
Ulcerative-colitis-grade-3	66	67	133
Total	5324	5338	10662

**Table 2:** Average DenseNet-161 and ResNet-152 results for both cross-validation splits. Best MCC score in bold.

Network	Resolution	MCC (R_k_)	F1-Score	Precision	Sensitivity
DenseNet-161	32 × 32	0.8241	0.5366	0.5414	0.5399
	64 × 64	0.8554	0.5701	0.5721	0.5748
	128 × 128	0.8865	0.6004	0.6033	0.6012
	256 × 256	0.8995	0.6149	0.6230	0.6141
	**512 × 512**	**0.9002**	0.6351	0.6446	0.6344
Resnet-152	32 × 32	0.8076	0.5108	0.5247	0.5137
	64 × 64	0.8556	0.5727	0.5725	0.5756
	128 × 128	0.8866	0.6112	0.6136	0.6137
	256 × 256	0.8965	0.6175	0.6182	0.6193
	**512 × 512**	**0.9002**	0.6115	0.6329	0.6171

**Table 3:** Average time for predicting output using DenseNet-161 and ResNet-152 in the inference stage.

	Time (ms) per Image	
Resolution	DenseNet-161	Resnet-152
32 × 32	19.875	17.190
64 × 64	20.248	15.148
128 × 128	21.606	15.450
256 × 256	20.246	14.986
512 × 512	20.422	16.690

## Data Availability

The dataset used for all experiments is publicly available at https://datasets.simula.no/hyper-kvasir (accessed on 16 April 2021).
